# Cardioprotective Effects of Dapagliflozin and Trimetazidine on Doxorubicin-Induced Cardiotoxicity in Streptozotocin-Induced Type 1 Diabetic Rats via Endoplasmic Reticulum Stress

**DOI:** 10.3390/jcm14041315

**Published:** 2025-02-16

**Authors:** Muhammed Mursel Ogutveren, Omer Satiroglu, Zulkar Ozden, Kerimali Akyildiz, Adnan Yilmaz, Filiz Mercantepe, Ahmet Seyda Yilmaz, Haldun Koc, Tolga Mercantepe

**Affiliations:** 1 Department of Cardiology, Faculty of Medicine, Recep Tayyip Erdogan University, 53100 Rize, Turkey; muhammedmursel.ogutveren@erdogan.edu.tr (M.M.O.); ahmetseyda.yilmaz@erdogan.edu.tr (A.S.Y.); halhaldun.koc@saglik.gov.tr (H.K.); 2 Department of Histology and Embryology, Faculty of Medicine, Recep Tayyip Erdogan University, 53100 Rize, Turkey; zulkar.ozden@saglik.gov.tr (Z.O.); tolga.mercantepe@erdogan.edu.tr (T.M.); 3 Department of Medical Services and Techniques, Health Services Vocational School, Recep Tayyip Erdogan University, 53100 Rize, Turkey; kerimali.akyildiz@erdogan.edu.tr; 4 Department of Biochemistry, Faculty of Medicine, Recep Tayyip Erdogan University, 53100 Rize, Turkey; adnan.yilmaz@erdogan.edu.tr; 5 Department of Endocrinology and Metabolism, Faculty of Medicine, Recep Tayyip Erdogan University, 53100 Rize, Turkey

**Keywords:** cardiotoxicity, diabetes mellitus, doxorubicin, endoplasmic reticulum stress, SGLT2-inhibitor, trimetazidine

## Abstract

**Background/Objectives:** Diabetic cardiomyopathy is a distinct myocardial dysfunction characterized by structural and functional changes in the heart that occur in diabetic patients independently of coronary artery disease or hypertension. It is closely associated with oxidative stress, inflammation, mitochondrial dysfunction, and endoplasmic reticulum (ER) stress, and contributes to progressive cardiac damage. This study aimed to evaluate the cardioprotective effects of dapagliflozin (DAPA) and trimetazidine (TMZ) in a rat model of doxorubicin-induced cardiomyopathy with streptozotocin-induced diabetes, focusing on their potential mechanisms related to ER stress. **Methods:** A total of 48 Sprague Dawley rats aged 6–8 weeks were randomly distributed equally into six cages. The diabetes model was induced by intraperitoneal administration of streptozotocin (STZ) and rats with blood glucose levels above 250 mg/dL were considered diabetic. For those rats with diabetes, cardiotoxicity was induced by intraperitoneal injection of 5 mg/kg/week doxorubicin (DOXO) for 4 weeks. After a cumulative dose of 20 mg/kg doxorubicin, a week break was given, followed by the administration of TMZ (10 mg/kg) and/or DAPA (10 mg/kg) to the treatment groups. **Results:** STZ administration caused diabetes and significant degeneration in cardiomyocytes. With the addition of DOXO (STZ + DOXO), cardiomyocyte degeneration became more severe. When the study groups were histopathologically evaluated based on parameters of degenerative cardiomyocytes, vascular congestion, and edema, it was shown that both TMZ and DAPA, whether applied alone or in combination, reduced damage in heart tissue. Both TMZ and DAPA reduced cardiomyocyte damage, and their combination provided the lowest level of damage through the reduced ER stress pathway by reducing GRP 78 and CHOP positivity. **Conclusions:** TMZ and DAPA reduce ER stress and have protective effects against diabetic-induced cardiotoxicity. Combination therapy or TMZ was found to be more effective than DAPA in alleviating ER stress. Combination therapy appears to carry potential effects for reducing cardiac cell damage in individuals with diabetes.

## 1. Introduction

Diabetes mellitus (DM) has become a serious public health issue with a rapidly increasing prevalence worldwide [1]. According to 2021 data, there are 537 million individuals with diabetes globally, and this number is projected to exceed 1.3 billion by 2050 [2]. A significant portion of morbidity and mortality in diabetic patients arises from diabetes-related complications [3]. Among adverse events, cardiovascular diseases are major causes of death and disability [4]. Diabetic cardiomyopathy (DCM) is a specific cardiac dysfunction that can develop in individuals with both type 1 and type 2 DM, with a 2–4 times higher risk of heart failure in diabetic patients compared with non-diabetic individuals [5]. In type 1 DM, structural and functional impairments in the heart muscle typically occur without the presence of coronary artery disease, hypertension, or other known cardiac causes, solely due to DM [6].

Multiple factors, including hyperglycemia, insulin resistance, oxidative stress, inflammation, fibrosis, and autonomic neuropathy, play a role in the development of DCM [7,8]. Emerging evidence suggests that even subclinical glycemic fluctuations, reflected by increased glycated hemoglobin (HbA1c) levels but not meeting the diagnostic threshold for diabetes, are associated with more severe subclinical cardiovascular dysfunction [9]. Therefore, a multifaceted approach that includes glycemic control, antioxidant therapies, insulin-sensitizing drugs, anti-inflammatory strategies, and lifestyle interventions is essential for the prevention and treatment of DCM [2,6,8,10].

Sodium-Glucose Co-Transporter-2 inhibitors (SGLT-2i) are a class of drugs widely used in recent years for the treatment of type 2 DM, which enhance glucose excretion by inhibiting glucose reabsorption in the kidneys [11]. Dapagliflozin (DAPA), functioning as an inhibitor of SGLT2, attenuates blood glucose concentrations through the blockade of glucose reabsorption within the renal system [12]. Nevertheless, the cardiovascular advantages of DAPA extend beyond mere glycemic regulation [13]. DAPA has the potential to safeguard cardiac tissue by diminishing oxidative stress and modulating the inflammatory response [14]. These beneficial effects were particularly evidenced in models of diabetes and cardiotoxicity [13,15,16,17,18]. In addition, trimetazidine (TMZ) is also another cardioprotective drug used for its anti-ischemic effects [16]. Its primary mechanism involves improving cellular energy metabolism and reducing oxidative stress-induced damage at the cellular level [17].

Doxorubicin (DOXO) is a potent antineoplastic agent in the anthracycline class that inhibits tumor growth by blocking DNA synthesis and replication [18]. It has proven efficacy in treating various cancers, including breast cancer, leukemia, lymphoma, sarcomas, and cancers of the bladder, lung, ovary, stomach, and thyroid [19]. The primary mechanisms of doxorubicin-induced cardiotoxicity include mitochondrial damage, oxidative stress, iron binding, and free radical production [20]. Malondialdehyde (MDA), a byproduct of lipid peroxidation, is widely used as a biomarker of oxidative stress, which can be used to evaluate cardiotoxicity secondary to DOXO [21]. Increased MDA levels in tissue and plasma indicate cellular membrane damage and free radical formation [22]. Glutathione (GSH) is also an essential component of the intracellular antioxidant defense system and contributes to the prevention of oxidative damage by neutralizing free radicals [23]. On the other hand, streptozotocin (STZ) damages pancreatic β-cells, reduces insulin production, and is widely used in experimental diabetes models [24]. By inducing a diabetes-like state, STZ causes hyperglycemia and serves as an essential tool in studying diabetes-related pathological processes [25].

Endoplasmic reticulum (ER) stress is postulated to significantly contribute to the pathophysiological mechanisms underlying both DCM- and doxorubicin-induced cardiotoxicity [26,27,28,29]. The elevation of glucose and free fatty acids in diabetic conditions results in an overload of proteins and disturbances in folding processes within the ER [30]. This condition subsequently activates the Unfolded Protein Response (UPR) pathway [30,31]. Nevertheless, the chronicity of diabetes can surpass the protective thresholds of the UPR, thereby inducing maladaptive responses such as apoptosis [32]. Furthermore, research indicates that ER stress initiates apoptotic pathways within the diabetic myocardium and exacerbates fibrosis and contractile dysfunction by amplifying the release of inflammatory cytokines [33]. Conversely, DOXO is recognized for its cardiotoxic properties notwithstanding its antitumor efficacy, and it is hypothesized that ER stress may be implicated in this detrimental process [26]. DOXO interferes with protein folding homeostasis by inducing the accumulation of reactive oxygen species (ROS) within the ER [34]. This phenomenon precipitates ER stress, and the unregulated activation of the UPR further exacerbates cellular injury. The ER stress induced by DOXO activates apoptotic mechanisms, predominantly via the C/EBP homologous protein (CHOP) [35]. The overexpression of CHOP instigates pro-apoptotic signaling pathways that exacerbate damage to cardiomyocytes [30]. CHOP subsequently mediates the downregulation of the anti-apoptotic protein Bcl-2 while promoting the expression of the pro-apoptotic protein Bax [26]. GRP 78 (Glucose-Regulated Protein 78) serves as a chaperone protein that exhibits upregulation in response to the ER stress and plays a crucial role in sustaining cellular homeostasis [36]. While it exerts an inactivating influence on sensor proteins, such as PERK, ATF6, and IRE1α under normative physiological conditions, it engages these sensors through the binding of misfolded proteins during ER stress, thereby initiating the UPR pathway [31]. At low to moderate levels of ER stress, the elevation of GRP 78 facilitates the adaptive response and mitigates the risk of apoptosis [37]. Nevertheless, in instances characterized by severe and protracted stress, the protective capacity of GRP 78 may become inadequate, potentially resulting in apoptotic processes [26]. When ER stress is alleviated, the expression levels of GRP 78 also diminish [26]. This observation signifies that the protein folding burden within the ER was alleviated, thereby indicating that the cell has attained a state of homeostatic equilibrium. Both DM and DOXO collaboratively enhance ER stress in conjunction with oxidative stress [26]. The accumulation of ROS further disrupts the protein folding milieu within the ER, instigating cellular dysfunction [36]. Pharmacological agents such as TMZ and DAPA may offer potential therapeutic benefits in these forms of cardiomyopathy by alleviating ER stress.

In consideration of the aforementioned data, the objective of the present investigation is to evaluate mediated by oxidative stress and ER stress pathways the impacts of DAPA, categorized as an SGLT2i, and TMZ, recognized as an anti-ischemic agent, on the cardiac tissue of rats subjected to DM through the administration of STZ and to cardiotoxicity through the application of DOXO. To achieve this, cardiac tissues from rats were subjected to both histopathological and biochemical analysis, in addition to being assessed using CHOP and GRP 78 immunohistochemical methodologies.

## 2. Materials and Methods

The research protocol received endorsement from the ethics committee of Recep Tayyip Erdogan University Animal Experiments Ethical Committee (Rize, Turkiye, Approval number: 2022/16 Approval Date: 26 April 2022). Upon completion of the study, all subjects were humanely euthanized utilizing high-dose anesthesia. Each phase of the research was conducted within the Experimental Animals Unit at Recep Tayyip Erdoğan University. The ARRIVE (Animal Research: Reporting In Vivo Experiments) guidelines were meticulously adhered to throughout all phases of the study [38].

### 2.1. Experimental Study Design

**Figure 1:**
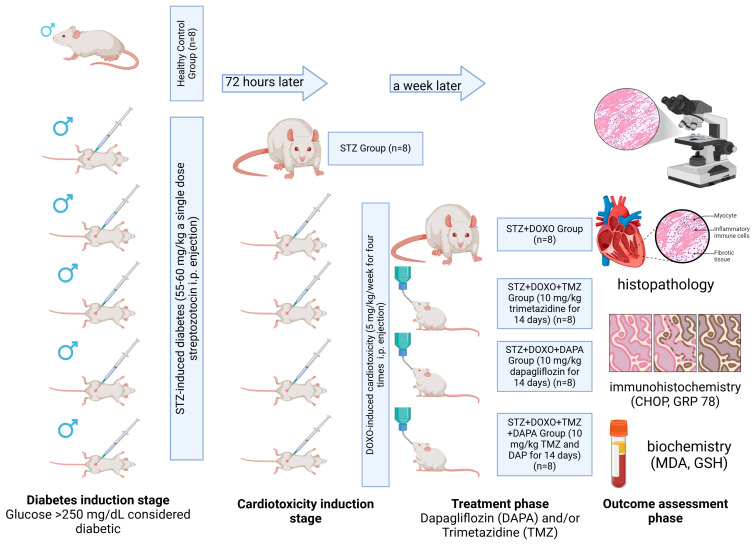
Schematic representation of experimental study design. Abbreviations: STZ, Streptozotocin; DOXO, Doxorubicin; TMZ, Trimetazidine; DAPA, Dapagliflozin; i.p., Intraperitoneal; CHOP, C/EBP Homologous Protein; GRP78, Glucose-Regulated Protein 78; MDA, Malondialdehyde; GSH, Glutathione.

The schematic representation of the experimental study design is illustrated in Figure 1. A cohort of forty-eight male Sprague Dawley rats, with an age range of six to eight weeks and an average body mass of 210 ± 30 g, was maintained in a regulated environment. This environment was characterized by a 12-hour light/dark cycle, and the rats were provided with unrestrained access to sustenance and water. The forty-eight rats were systematically distributed into six equal groups through a computerized randomization procedure, executed by a researcher who was blinded to the specific group allocations. The determination of sample size was informed by the existing literature research [39].

The study groups were designed as described below.

Group 1 (Control, *n* = 8): No external intervention was applied to the rats in this group.

Group 2 (STZ, *n* = 8): A diabetes model was created by administering a single dose of 55–60 mg/kg intraperitoneal (i.p.) streptozotocin to the rats in this group [24]. It was considered that diabetes induction was achieved when the glucose value measured by a glucometer from the tail blood of the rats was >250 mg/dL 72 h after STZ administration [24].

Group 3 (STZ + DOXO, *n* = 8): After it was proven that diabetes was induced in the rats in this group with STZ, cardiotoxicity was induced with 5 mg/kg i.p. doxorubicin once a week for 4 weeks. The cumulative dose of doxorubicin was 20 mg/kg [40].

Group 4 (STZ + DOXO + TMZ, *n* = 8): After inducing diabetes with STZ and cardiotoxicity with DOXO to the rats in this group, TMZ was administered orally at a dose of 10 mg/kg for 14 days, starting 1 week after the last DOXO dose [41].

Group 5: (STZ + DOXO + DAPA, *n* = 8): After inducing diabetes with STZ and cardiotoxicity with DOXO in the rats in this group, DAPA was administered orally at a dose of 10 mg/kg for 14 days, starting 1 week after the last DOXO dose [42].

Group 6: (STZ + DOXO + TMZ + DAPA, *n* = 8): After inducing diabetes with STZ and cardiotoxicity with DOXO to the rats in this group, TMZ was administered orally at a dose of 10 mg/kg and DAPA was administered at a dose of 10 mg/kg for 14 days, starting 1 week after the last DOXO dose.

The diabetes model was induced by intraperitoneal administration of STZ. A single dose of 55–60 mg/kg STZ was dissolved in freshly prepared 0.1 M citrate buffer at pH 4.5 and administered [24]. Seventy-two hours after STZ injection, fasting blood glucose levels were measured from tail vein samples using a glucometer (On Call Plus, Acon Laboratories Inc., 10125 Mesa Rim Road, San Diego, CA 92121, USA). Rats with fasting blood glucose levels exceeding 250 mg/dL were considered diabetic [24]. At the end of the experiment (14th day of TMZ and DAPA administration), all subjects were euthanized using high-dose anesthetics (50 mg/kg ketamine HCl and 10 mg/kg xylazine HCl), and the required blood and tissue samples were collected. The heart tissue removed from the rats sacrificed under anesthesia was washed with 0.9% cold (+4 °C) NaCl solution and dried with filter paper, then divided into two equal parts in the coronal plane. One part was placed in a 10% formaldehyde solution for histopathological and immunohistochemical examinations. The other part was stored in portions at −80 °C until further analysis to be used in biochemical analyses. The specimens were procured in a manner that would facilitate a comprehensive assessment of all anatomical components of the cardiac tissue.

### 2.2. Outcomes

The primary outcomes of this study included histopathological, immunohistochemical, and biochemical markers of cardiotoxicity as detailed below.

- Histopathological outcomes: Cardiomyocyte degeneration, vascular congestion, and edema (evaluated using histopathological cardiac damage scoring, HCDS).
- Immunohistochemical outcomes: CHOP and GRP 78 positivity in cardiomyocytes (markers of ER stress).
- Biochemical outcomes: Malondialdehyde (TBARS assay as MDA, marker of oxidative stress) and Glutathione (Total thiol, GSH, antioxidant marker).
- Mediator Variables (Mechanistic Pathways Tested):
- ER Stress Pathway: Evaluated using CHOP and GRP 78 immunohistochemistry.
- Oxidative Stress Pathway: Assessed via MDA and GSH levels biochemically.

### 2.3. Biochemical Analysis

#### 2.3.1. Homogenization of Heart Tissues

A homogenization buffer was prepared with 20 mM sodium phosphate and 140 mM potassium chloride (pH 7.4). The heart tissues were washed in cold PBS buffer (pH 7.4) to remove excess water and then weighed. The homogenization buffer was added to the heart tissues at a tissue weight (g) to PBS volume (mL) ratio of 1:9, and the tissues were homogenized using a QIAGEN Tissue Lyser II homogenizer. The homogenates were then centrifuged at 800× *g* for 10 min at +4 °C. The resulting supernatants were used for the determination of Thiobarbituric Acid Reactive Substances (TBARS) and total thiol (TT) groups.

#### 2.3.2. Thiobarbituric Acid Reactive Substances (TBARS) Assay

The TBARS assay was performed according to the modified method of Ohkawa et al. [43]. The principle of the method is based on the formation of a pink-colored complex when the lipid peroxidation product MDA was heated with a thiobarbituric acid (TBA) solution. From the standards and samples, 200 µL was pipetted, and 50 µL of 8.1% SDS, 375 µL of 20% acetic acid (pH 3.5), and 375 µL of 0.8% TBA were added. The mixture was vortexed and then incubated in a boiling water bath for 1 h. After incubation, the samples were cooled in ice-cold water and centrifuged at 750× *g* for 10 min. The resulting pink color was measured at 532 nm using a spectrophotometer, and a standard curve was plotted to calculate concentrations based on the standard graph. The results were expressed as nmol/g tissue.

#### 2.3.3. Determination of Total Thiol (TT) Groups

The determination of the total thiol groups was based on the spectrophotometric measurement of the yellow color formed by free sulfhydryl groups reacting with Ellman’s reagent [44]. A volume of 25 µL from the standards and samples was pipetted, followed by the addition of 100 µL 3 M Na_2_HPO_4_ and 25 µL DTNB. After gently mixing, the resulting yellow color was measured at 412 nm using a spectrophotometer. The results were expressed as mmol/g tissue.

### 2.4. Histopathological Analysis

Heart tissue samples obtained from male Sprague Dawley rats were sectioned into pieces of 1.5 cm^3^. The samples were fixed by immersing them in a 10% phosphate-buffered formalin solution (Sigma Aldrich, München, Germany) for 24–36 h. Following fixation, routine histological procedures were performed, including dehydration (using an ascending ethanol series, Merck KGAa, Darmstadt, Germany), clearing (with xylene, Merck KGAa, Darmstadt, Germany), embedding in soft paraffin (Merck KGAa, Darmstadt, Germany), and finally blocking with hard paraffin (Merck KGAa, Darmstadt, Germany). Then, 4–5 µm thick sections were taken from the paraffin blocks of the heart tissue separated for histopathological analyses using a rotary microtome (Leica RM2525, Leica, Nussloch, Germany) and stained with Harris Hematoxylin and Eosin G (H&E; Merck KGAa, Darmstadt, Germany). All histopathological examinations were performed independently by two histologists using an Olympus trinocular BX51 TF microscope (Olympus, Tokyo, Japan) equipped with an Olympus DP74 camera attachment.

### 2.5. Immunohistochemical Analysis Procedure

CHOP (ab3392, Abcam, Cambridge, UK) and GRP 78 (ab21685, Abcam, Cambridge, UK) primary antibodies were used to identify endoplasmic reticulum stress. Additionally, a secondary antibody kit compatible with the primary antibodies (Goat Anti-Rabbit IgG H&L (HRP), ab205718, Abcam, Cambridge, UK) was utilized.

Sections of 1–2 µm thickness were obtained from the heart tissue samples, followed by deparaffinization according to the manufacturer’s instructions. Subsequently, antigen retrieval procedures were performed. The immunohistochemical staining was conducted using a Bond Max II IHC/ISH staining device (Leica Biosystems, Nussloch, Germany), with the sections incubated for 60 min with primary and secondary antibodies. In the next step, the heart tissue sections were stained with diaminobenzidine tetrahydrochloride (Ultraview, Leica Biosystems, Nussloch, Germany) and Harris hematoxylin (Merck KGAa, Darmstadt, Germany).

### 2.6. Semi-Quantitative Analysis

In the present investigation, the assessment of cardiac structural impairment was grounded upon parameters that are routinely employed in histopathological evaluations and are delineated within the literature. The parameters under consideration include degenerative cardiomyocytes, vascular congestion, and edema [45]. Degenerative cardiomyocytes were examined based on alterations in cellular morphology. Such alterations encompass cytoplasmic vacuolization, pyknotic nuclei, cellular edema, and the loss of myofibrils. These observations signify that cardiomyocytes sustain damage, leading to compromised cellular functionality. Vascular congestion is characterized as the dilation and engorgement of blood vessels within cardiac tissue [45]. This phenomenon frequently signifies compromised tissue perfusion and irregularities within microcirculation. Edema within cardiac tissue is characterized as the accumulation of fluid in the interstitial compartments and serves as an early indicator of tissue inflammation or cellular injury [45]. Microscopic assessment reveals edema through the expansion of intercellular spaces coupled with the aggregation of interstitial fluid. Edema is typically correlated with the intensity of inflammatory processes and was frequently noted in studies of doxorubicin-induced cardiotoxicity [41,42,46]. In the current study, the aforementioned parameters were scrutinized utilizing H&E staining, and severity was classified for each parameter (0 = none = ≤5%; 1 = mild = 6–25%; 2 = moderate = 26–50%; 3 = severe = >50%) (Table 1) [41,42,46,47,48,49]. This methodology represents a well-established approach in the literature and facilitates a quantitative evaluation of cardiac damage [41,42,45,46]. For 10 sections of cardiac tissue, 25 distinct regions chosen at random were meticulously assessed at 40× magnification by two independent histopathologists who were blinded to the study groups.

Immuno-positive cardiomyocytes for CHOP and GRP 78 primary antibodies were also analyzed by a double-blinded histopathologist for the study groups, as shown in Table 2. In each of the 10 slides, 25 randomly selected areas were examined to measure IHC positivity.

### 2.7. Statistical Analysis

A priori sample size estimation was performed using GPower 3.1 software. The calculation was based on the expected differences in histopathological cardiac damage scoring (HCDS) between groups, as this was considered an important primary outcome in assessing cardiotoxicity. A two-tailed alpha (α) of 0.05, a power (1 − β) of 0.80, and an effect size (Cohen’s d) of 1.2 were used. Based on this estimate, a minimum of six animals per group was determined to be sufficient. To account for potential variability and attrition, eight animals per group were included in the study. All collected data were complete, and there were no missing data on any variables in this study. The data were statistically analyzed using the SPSS 21.0 software. The appropriateness of the biochemical, histopathological, and immunohistochemical data for normal distribution was evaluated using the Shapiro–Wilk test, Skewness–Kurtosis values, Q-Q plots, and Levene’s test. Parametric data were expressed as mean ± standard deviation, while non-parametric data were expressed as median and interquartile range (25–75%). The One-Way ANOVA test was used for comparisons of continuous variables with more than two independent groups since the normal distribution condition was met. Non-parametric data were also analyzed by Kruskal–Wallis test. Post hoc tests after One-Way ANOVA or Kruskal–Wallis test were performed using Bonferroni test when variances were homogeneous and Tamhane’s T2 test when variances were not homogeneous. A *p*-value of <0.05 was considered statistically significant.

## 3. Results

### 3.1. Biochemical Analysis

No statistically significant differences were found in the tissue MDA and GSH levels among the STZ, STZ + DOXO, STZ + DOXO + TMZ, STZ + DOXO + DAPA, and STZ + DOXO + TMZ + DAPA groups compared with the control group (*p* > 0.05) (Table 3).

### 3.2. Histopathological Analysis

**Figure 2:**
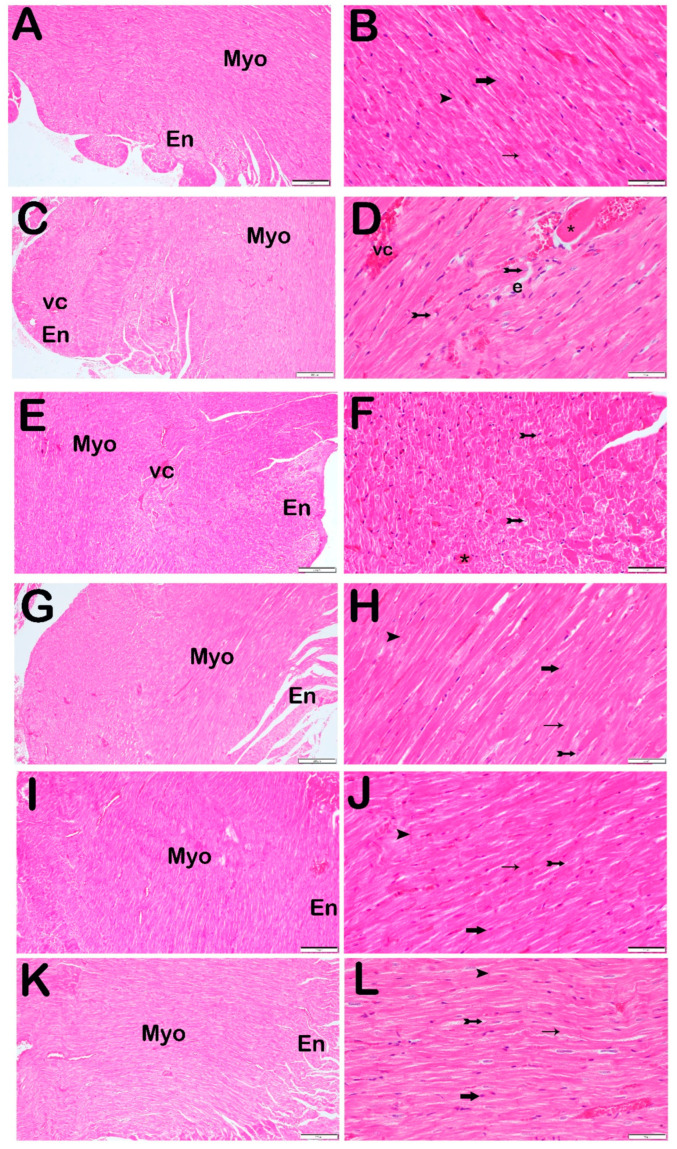
Representative light microscopic images of the heart tissue sections stained with H&E. Endocardium (En), Myocardium (Myo), Vascular Congestion (vc), cardiac amyloidosis (asterisk), Edema (e). (**A**) (×10) and (**B**) (×40) Control Group: When sections of the heart tissue were examined under a light microscope, it was observed that there are cardiomyocytes (arrow) containing normal structured bands (thin arrow) and intercalary disk (arrowhead) in the normal myocardium layer (HCDS median: 0 (0–1)). (**C**) (×10) and (**D**) (×40) STZ Group: In the heart tissue sections of the STZ application group, widespread degenerative cardiomyocytes (arrow with a tail), vascular congestion (vc), cardiac amyloidosis (asterisk) and edematous areas (e) were observed (HCDS median: 5 (5–5)). (**E**) (×10) and (**F**) (×40): STZ + DOXO Treatment Group: When sections of the heart tissue were examined under a light microscope, degenerative cardiomyocytes (arrow with a tail), vascular congestion (vc), cardiac amyloidosis (asterisk) and edematous areas (e) were observed (HCDS median: 6 (5–6)). (**G**) (×10) and (**H**) (×40): STZ + DOXO + TMZ Treatment Group: When sections of the heart tissue were examined under a light microscope, degenerative cardiomyocytes (arrow with a tail), vascular congestion, and edematous areas were observed to be reduced (HCDS median: 3 (3–4)). (**I**) (×10) and (**J**) (×40): STZ + DOXO + DAPA Treatment Group: Although cardiomyocytes with a widespread typical structure were observed in sections of the heart tissue, degenerative cardiomyocytes were observed to be reduced (HCDS median: 3 (3–3)). (**K**) (×10) and (**L**) (×40): STZ + DOXO + DAPA Treatment Group: When sections of the heart tissue were examined under a light microscope, degenerative cardiomyocytes, vascular congestion, and edematous areas were observed to be reduced. In addition, typical cardiomyocytes (arrow) were observed in sections of the heart tissue (HCDS median: 3 (3–2)).

When sections of the heart tissue stained with Harris Hematoxylin and Eosin G were examined under a light microscope, normal-structured cardiomyocytes were observed in the normal myocardium layer of the control group (Figure 2A,B, Table 4, *p* = 0.001, HCDS: 0 (0–1)). In the heart tissue sections from the STZ group, widespread degenerative cardiomyocytes, extensive edematous areas, and vascular congestion were observed in the myocardium layer compared with the control group (Figure 2C,D, Table 4, *p* = 0.001, HCDS: 5 (5–5)). Similarly, in the heart tissue sections from the STZ + DOXO group, extensive degenerative cardiomyocytes, edematous areas, and vascular congestion was observed in the myocardium layer compared with the control group (Figure 2E,F, Table 4, *p* = 0.001, HCDS: 6 (5–6)). Furthermore, in the STZ + DOXO group, an increase in degenerative cardiomyocytes (degenerative cardiomyocyte damage scores: 2 (2–2) vs. 3 (2–3), Table 4, *p* = 0.001) and vascular congestion (degenerative cardiomyocyte damage scores: 2 (2–2) vs. 3 (2–3), Table 4, *p* = 0.001) was detected compared with the STZ group (Figure 2C–F, *p* = 0.001, HCDS: 6 (5–6)).

In contrast, in the STZ + DOXO + TMZ group, a reduction in extensive degenerative cardiomyocytes, edematous areas, and vascular congestion in the myocardium layer was observed compared with the STZ and STZ + DOXO groups (Figure 2G,H, Table 4, *p* = 0.001, HCDS: 3 (3–4)). Similarly, in the STZ + DOXO + DAPA group, extensivity of degenerative cardiomyocytes, edematous areas, and vascular congestion in the myocardium layer was reduced compared with the STZ and STZ + DOXO groups (Figure 2I,J, Table 4, *p* = 0.001, HCDS: 3 (3–3)). In the STZ + DOXO + TMZ + DAPA group, extensivity of degenerative cardiomyocytes, edematous areas, and vascular congestion was reduced compared with the STZ and STZ + DOXO groups, and the myocardium layer displayed general typical cardiomyocytes (Figure 2K,L, Table 4, *p* = 0.005, HCDS: 3 (3–3)).

### 3.3. Immunohistochemical Analysis

#### 3.3.1. CHOP Primary Antibody

**Figure 3:**
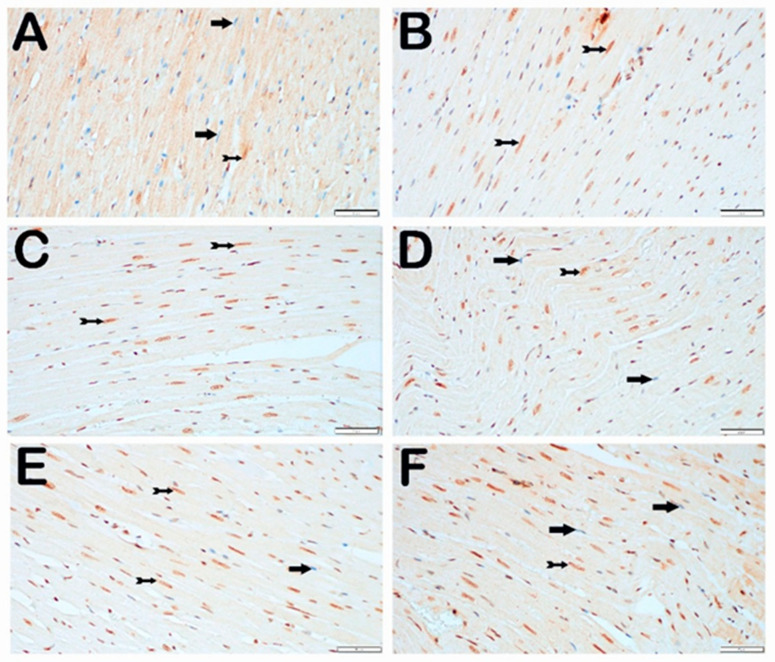
Representative light microscopic images of the heart tissue sections stained with CHOP primary antibody. Immuno-negative cardiomyocytes (arrow), immuno-positive cardiomyocytes (arrow with a tail). (**A**) (×40) Control Group: An examination of the heart tissue sections under a light microscope revealed that normal cardiomyocytes (arrow) were immuno-negative for the CHOP primary antibody (CHOP positivity score: 0 (0–0)). (**B**) (×40) STZ Group: In the heart tissue sections, an increase in the number of cardiomyocytes (arrow with a tail) exhibiting strong CHOP positivity was observed (CHOP positivity score: 1 (1–2)). (**C**) (×40) STZ + DOXO Group: An increase in the number of cardiomyocytes (arrow with a tail) with strong CHOP positivity in the myocardium was noted (CHOP positivity score: 2 (2–2)). (**D**) (×40) STZ + DOXO + TMZ Group: A reduction in the number of CHOP-positive cardiomyocytes was observed (CHOP positivity score: 1 (1–1)). (**E**) (×40) STZ + DOXO + DAPA Group: A decrease in the number of cardiomyocytes with strong CHOP positivity in the myocardium was noted (arrow with a tail) (CHOP positivity score: 1 (1–1)). (**F**) (×40) STZ + DOXO + TMZ + DAPA Group: A reduction in the number of cardiomyocytes with strong CHOP positivity in the myocardium was observed, along with widespread immuno-negative cardiomyocytes (arrow) (CHOP positivity score: 1 (1–1)).

When endoplasmic reticulum stress was evaluated using the IHC method with CHOP primary antibody incubation, cardiomyocytes with normal structure in the control group were observed to be immuno-negative for the CHOP primary antibody (Figure 3A; Table 5; CHOP positivity score: 0 (0–1)). In contrast, in the STZ group, a significant increase in the number of cardiomyocytes demonstrating strong CHOP immuno-positivity was observed compared with the control group (Figure 3B; Table 5, *p* = 0.001, CHOP positivity score: 1 (1–2)). Similarly, in the STZ + DOXO group, the number of cardiomyocytes demonstrating strong immuno-positivity increased compared with the control group (Figure 3C; Table 5, *p* = 0.001, CHOP positivity score: 2 (2–2)).

Furthermore, an increase in the number of cardiomyocytes with strong immuno-positivity was observed in the STZ + DOXO group compared with the STZ group (Figure 3B,C; Table 5, *p* = 0.001; CHOP positivity scores: 1 (1–2) vs. 2 (2–2), respectively). In the STZ + DOXO + TMZ group, a reduction in the number of CHOP-positive cardiomyocytes was observed compared with the STZ and STZ + DOXO groups (Figure 3D; Table 5, *p* = 0.001, CHOP positivity score: 1 (1–1)). Similarly, in the STZ + DOXO + DAPA group, a decrease in the number of strongly CHOP-positive cardiomyocytes was observed (Figure 3E; Table 5, *p* = 0.001, CHOP positivity score: 1 (1–1)). Finally, in the STZ + DOXO + TMZ + DAPA group, the number of CHOP-positive cardiomyocytes significantly decreased compared with the STZ and STZ + DOXO groups (Figure 3F; Table 5, *p* = 0.001, CHOP positivity score: 1 (1–1)).

#### 3.3.2. GRP 78 Primary Antibody

**Figure 4:**
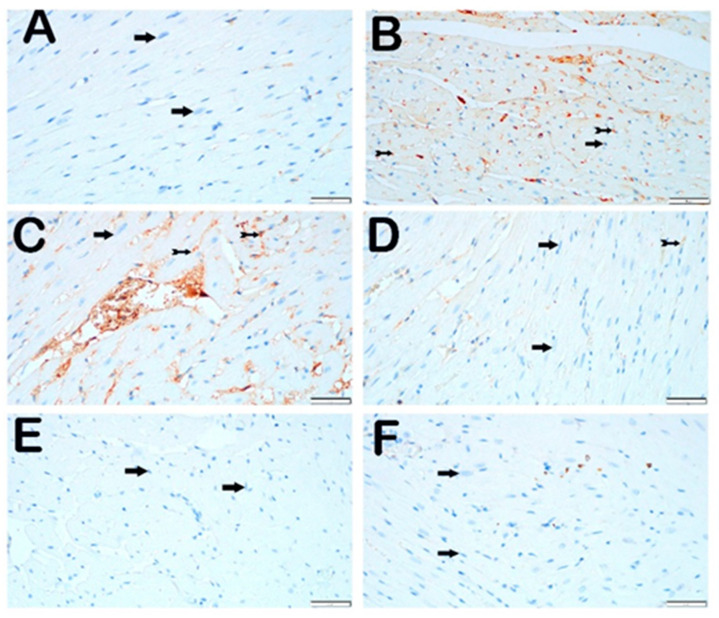
Representative light microscopic images of the heart tissue sections staining with GRP 78 primary antibody. Immuno-negative cardiomyocytes (arrow), immuno-positive cardiomyocytes (arrow with a tail). (**A**) (×40) Control group: When the heart tissue sections were examined under a light microscope, cardiomyocytes with normal structure (arrow) that are immuno-negative for GRP 78 primary antibody are observed (GRP 78 positivity score: 0 (0–0)). (**B**) (×40) STZ group: When the heart tissue sections were examined under a light microscope, it was observed that cardiomyocytes showing intense GRP 78 positivity (arrow with a tail) increased in number (GRP 78 positivity score: 1 (1–2)). (**C**) (×40) STZ + DOXO group: It was observed that cardiomyocytes positive for intense GRP 78 primary antibody (arrow with a tail) were increased (GRP 78 positivity score: 1 (1–2)). (**D**) (×40) STZ + DOXO + TMZ group: The number of cardiomyocytes positive for GRP 78 primary antibody appears to be reduced. Cardiomyocytes positive for GRP 78 primary antibody are immuno-negative (arrow with a tail) and decreased cardiomyocytes were observed (GRP 78 positivity score: 0 (0–1)). (**E**) (×40) STZ + DOXO + DAPA group: It was observed that cardiomyocytes with intense GRP 78 staining decreased (GRP 78 positivity score: 1 (0–1)). (**F**) (×40) STZ + DOXO + TMZ + DAPA group: It was observed that cardiomyocytes with intense GRP 78 staining decreased (GRP 78 positivity score: 0 (0–1)).

When endoplasmic reticulum stress was evaluated using the IHC method with GRP 78 primary antibody incubation, cardiomyocytes with normal structure in the control group were observed to be negative for GRP 78 (Figure 4A; Table 5; GRP 78 positivity score: 0 (0–0)). In the STZ group, an increase in the number of cardiomyocytes showing strong GRP 78 positivity was observed compared with the control group (Figure 4B; Table 5, *p* = 0.001, GRP 78 positivity score: 1 (1–2)). Similarly, in the STZ + DOXO group, an increase in the number of cardiomyocytes with strong immuno-positivity was observed compared with the control group (Figure 4C; Table 5, *p* = 0.001, GRP 78 positivity score: 1 (1–2)).

In the STZ + DOXO + TMZ group, a reduction in the number of GRP 78-positive cardiomyocytes was observed compared with the STZ and STZ + DOXO groups (Figure 4D; Table 5, *p* = 0.001, GRP 78 positivity score: 0 (0–1)). Similarly, in the STZ + DOXO + DAPA group, a decrease in the number of GRP 78-positive cardiomyocytes was observed (Figure 4E; Table 5, *p* = 0.001, GRP 78 positivity score: 1 (0–1)). In the STZ + DOXO + TMZ + DAPA group, a significant reduction in the number of GRP 78-positive cardiomyocytes was detected compared with the STZ and STZ + DOXO groups (Figure 4F; Table 5, *p* = 0.001, GRP 78 positivity score: 0 (0–1)).

## 4. Discussion

In the present study, the effects of dapagliflozin and trimetazidine on ER stress were evaluated in a rat model where cardiotoxicity was induced by doxorubicin and diabetes was induced by streptozotocin. Although changes in MDA and GSH levels were observed among the groups, no statistically significant differences were detected. However, the administration of trimetazidine and dapagliflozin restored the histological structure of the heart, and IHC analyses confirmed these agents’ abilities to reduce ER stress.

In the progress of diabetic complications, factors such as oxidative stress, hyperlipidemia, fibrosis progression, and insulin resistance, in addition to inflammation, play a significant role [50]. Chronic hyperglycemia in diabetic patients leads to metabolic dysfunction and the accumulation of advanced glycation end-products [51]. This, in turn, causes disruptions in cellular energy metabolism, oxidative stress in cardiomyocytes, fibrosis in cardiac muscle, and ultimately cardiac dysfunction [52]. Moreover, hyperglycemia in diabetic patients impairs mitochondrial function, affecting energy production, which results in energy deficits in cardiomyocytes and compromises cardiac functions [10]. Additionally, insulin resistance in diabetic patients inhibits the utilization of glucose by cardiomyocytes for energy production, leading to the use of fatty acids as an energy source. This process results in the accumulation of lipids in cardiomyocytes, causing lipotoxicity [8]. Furthermore, hyperglycemia increases the production of ROS, weakens antioxidant defense systems, and thus leads to protein, lipid, and DNA damage in cardiomyocytes, culminating in cell death and tissue damage [53]. Hyperglycemia also promotes fibrosis in cardiomyocytes by activating fibroblasts and increasing the production of extracellular matrix proteins such as collagen [7]. Pathways such as transforming growth factor-β (TGF-β), which play a key role in fibrosis progression, are upregulated in diabetic individuals [2]. Thus, chronic hyperglycemia is associated with a chronic inflammatory state. During inflammation, levels of proinflammatory cytokines such as tumor necrosis factor (TNF)-α, interleukin (IL)-6, and IL-1β increase, which trigger apoptosis and fibrosis in cardiomyocytes and contribute to endothelial dysfunction [45], are increased.

Malondialdehyde, the end-product of lipid peroxidation, is a significant marker of oxidative damage [21,43]. The increase in MDA levels in the group treated with STZ-induced diabetes confirms that diabetes elevates oxidative stress. In our study, MDA levels in the group treated with STZ combined with DOXO were found to be similar. This suggests that doxorubicin induces cardiotoxicity through mechanisms other than oxidative stress. TMZ was shown to improve left ventricular function, exercise capacity, and cardiac energy efficiency in diabetic cardiomyopathy is generally attributed these effects through mechanisms such as inhibiting fatty acid oxidation, improving mitochondrial function, reducing oxidative stress, and preventing cellular acidosis [54]. In our study, TMZ administration brought MDA levels closer to those of the control group, although it was not statistically significant. This result highlights the protective potential of TMZ against oxidative stress. On the other hand, SGLT-2 inhibitors were proven to provide cardiovascular protection in both diabetic and non-diabetic heart failure patients [55]. The beneficial effects of SGLT-2 inhibitors are associated with various mechanisms independent of diabetes, including diuresis, natriuresis, hemodynamic effects, reduction in inflammation, oxidative stress, fibrosis, and uric acid levels, as well as the utilization of alternative energy sources in the heart [12,56]. In our study, a slight reduction in MDA levels was observed in the groups treated with dapagliflozin, an SGLT-2 inhibitor. This finding suggests that the cardioprotective effects of dapagliflozin are likely mediated through mechanisms other than oxidative stress.

GSH levels are well known to be an indicator of cellular antioxidant capacity [23,44]. In the current study, GSH levels did not show significant differences among the groups. However, slightly higher GSH levels were observed in the groups treated with TMZ. This suggests that TMZ may play a supportive role in enhancing antioxidant protections. Studies in the literature report that TMZ protects heart tissue against oxidative damage and that dapagliflozin has positive effects on antioxidant defense systems [57,58,59]. In our study, the effect of TMZ on oxidative stress was found to be more prominent compared to dapagliflozin, and when the study groups were histopathologically evaluated based on parameters such as degenerative cardiomyocytes, vascular congestion, and edema, it was observed that the administration of TMZ and dapagliflozin, both individually and in combination, reduced cardiac tissue damage. These findings suggest that TMZ may be a more effective therapeutic option against cardiotoxicity compared to dapagliflozin. However, since cardioprotective effects are not solely mediated through oxidative stress, further investigation is warranted to better understand the reduced effectiveness of dapagliflozin or combined therapy in addressing oxidative stress. Trimetazidine is known to exert cardioprotective effects by regulating energy metabolism and reducing oxidative stress [60]. Dapagliflozin, on the other hand, functions as an agent that reduces cardiac load and stabilizes glucose levels [13]. The combination of these two agents is thought to have assisted with minimizing the degeneration observed in cardiac tissue.

Doxorubicin, although a potent chemotherapeutic agent widely used in cancer treatments, has severe side effects, including cardiotoxicity, which can lead to heart failure [20,61]. The primary mechanisms of doxorubicin-induced cardiotoxicity include mitochondrial damage, oxidative stress, iron binding, free radical production, and apoptosis [20]. To prevent doxorubicin-induced cardiotoxicity, various approaches such as low-dose strategies, protective agents like anti-inflammatories and antioxidants, liposomal doxorubicin, dexrazoxane, ACE inhibitors, and beta-blockers were developed [41]. However, none have provided a complete solution. In our study, vascular congestion in the heart was more noticeable, particularly in the groups treated with doxorubicin, which can be considered an indicator of microvascular damage due to cardiotoxicity. Treatment with TMZ and dapagliflozin significantly reduced vascular congestion, with this effect being more obvious in combination therapy. Cardiac edema, another important indicator of cardiac dysfunction, was markedly reduced in the groups treated with TMZ and dapagliflozin, especially in combination therapy, which decreased edema to a minimal level. The vascular protective effects of TMZ and dapagliflozin manifested through the reduction in vascular congestion and edema in cardiac tissue. The reduction in vascular congestion can be attributed to TMZ’s anti-ischemic effects, while the reduction in edema may be associated with the anti-inflammatory properties of dapagliflozin. These findings suggest that both agents provide cardioprotection through different mechanisms at the vascular and cellular levels. In light of the constraints inherent to our investigation, a conclusive determination regarding the synergistic or individual effects of dapagliflozin and trimetazidine on the attenuation of ER stress remains elusive. Nonetheless, the existing literature elucidates that both pharmacological agents demonstrate efficacy in mitigating ER stress via distinct mechanisms [26,29]. These observations furnish a significant foundation for prospective research endeavors, and the application of sophisticated molecular methodologies is imperative to explore the potential existence of a synergistic effect arising from the concomitant administration of these two therapeutic agents.

Trimetazidine, as widely recognized in the literature, enhances glucose utilization in cellular contexts by inhibiting the process of mitochondrial beta-oxidation [62]. This particular mechanism may yield favorable outcomes in the enhancement of cardiac function by modulating energy metabolism in pathological conditions such as cardiomyopathy [17,54,62]. Nevertheless, mitochondrial dysfunction was not directly assessed within the parameters of the present study. Rather, the cardioprotective properties of trimetazidine were scrutinized through histopathological and biochemical methodologies, with a specific focus on the pathways associated with endoplasmic reticulum stress and oxidative stress. This represents an indirect methodology for evaluating the influence of trimetazidine on mitochondrial functionality. Mitochondrial dysfunction constitutes a critical element in the pathophysiology of cardiomyopathy, and a more comprehensive exploration of the impact of trimetazidine within this domain may represent a significant avenue for future investigative endeavors [8]. Particularly, a more thorough investigation of these mechanisms utilizing parameters that directly measure mitochondrial functions (such as mitochondrial ATP production, levels of oxidative stress, or mitochondrial membrane potential) could advance the understanding of the cardioprotective effects attributed to trimetazidine.

The present study also focuses on the effects of ER stress on diabetes and doxorubicin-induced cardiotoxicity. ER stress plays a critical role in conditions of disrupted cellular homeostasis, particularly in cardiomyocytes [63]. The status of ER stress markers, GRP 78 and CHOP, in cardiac tissue was investigated immunohistochemically. GRP 78 is a molecule that increases in response to ER stress and reflects protein-folding stress within the cell. CHOP, on the other hand, is a molecule that triggers apoptotic pathways during the advanced stages of ER stress [26]. In this study, streptozotocin and doxorubicin administration significantly increased the levels of GRP 78 and CHOP, indicating that cellular stress associated with diabetes and cardiotoxicity is exacerbated via the ER pathway. It was previously reported that TMZ suppresses ER stress-related apoptosis and regulates GRP 78 expression in cardiomyocytes [64]. Furthermore, trimetazidine is an agent that regulates cellular energy metabolism and reduces oxidative stress [62]. This agent may contribute to alleviating ER stress by optimizing ATP production and mitochondrial functions [29,62]. Similarly, dapagliflozin has the potential to preserve cardiac functions through its anti-inflammatory and antioxidant effects and may also alleviate ER stress [26]. In this study, TMZ and dapagliflozin were shown to alleviate ER stress by reducing both GRP 78 and CHOP positivity. However, TMZ and combination therapy provided a more pronounced reduction compared to dapagliflozin alone. These findings suggest that TMZ and dapagliflozin have beneficial effects on ER stress markers, both independently and in combination. Notably, trimetazidine alone and in combination therapy were found to be more effective than dapagliflozin therapy. This indicates that the protective effects of dapagliflozin against ER stress may be more limited compared to agents that directly target energy metabolism, with TMZ offering more robust protection against ER stress.

On the other hand, an important aspect of our study is that it highlights the potential benefits of combining trimetazidine and dapagliflozin in cardiotoxicity induced by both diabetes and doxorubicin. In the future, the combined use of these two agents in clinical settings where cardiotoxicity due to diabetes and other causes coexist may be effective in reducing the risk of cardiotoxicity, particularly in diabetic patients. This study may pave the way for the development of new therapeutic strategies for clinical conditions where diabetes and cardiotoxicity are concurrently observed. Additionally, by investigating the effects of TMZ and dapagliflozin through more specific molecular pathways, the mechanisms of action of these agents on cardiac tissue can be better understood. Beyond GRP 78 and CHOP, other markers of ER stress, such as XBP1 and ATF6, should also be examined to provide a broader molecular mechanism perspective [65]. This would enable a better understanding of the effects of TMZ and dapagliflozin on other pathways regulating ER stress. The cardioprotective properties of dapagliflozin and trimetazidine extend beyond ER stress, as evidenced by the existing literature that indicates these pharmacological agents are implicated in various mechanisms, including the attenuation of oxidative stress, enhancement of mitochondrial functionality, regulation of energy metabolism, and modulation of inflammatory pathways [26,29]. Specifically, it is reported that these agents may mitigate damage to cardiomyocytes by inhibiting oxidative stress, diminishing mitochondrial biogenesis, and curtailing inflammation [26,29,66]. In this regard, it is imperative to emphasize that alongside the ER stress responses examined in our investigation, further rigorous experimental studies are warranted to clarify the interrelations and potential synergistic effects of these mechanisms.

While our study has limitations, it also has notable strengths. It is among the few examining the combined cardioprotective effects of dapagliflozin and trimetazidine in a diabetic cardiotoxicity model. Using histopathological, immunohistochemical, and biochemical analyses, we provided mechanistic insights into their effects. Blinded outcome assessments enhanced reliability, and the study followed randomized controlled procedures and ARRIVE guidelines, ensuring methodological rigor. Our findings support the potential clinical benefits of these drugs in diabetic cardiotoxicity treatment. Despite these strengths, certain limitations should be acknowledged to provide a balanced interpretation of our findings. This study was conducted with a relatively small number of subjects. The wide variation observed in the parameters studied in small sample size studies posed a limitation, negatively affecting the interpretation of our biochemical results. Furthermore, the study was performed on subjects with a short follow-up period. Additionally, this study examined ER stress only through GRP 78 and CHOP. Biochemically, only MDA and GSH parameters were examined. The weight of the cardiac tissue was not documented. The study was designed to induce cardiotoxicity solely through streptozotocin and doxorubicin. At the end of the experimental procedures, the measurement of blood glucose levels was not executed, nor was there an assessment conducted to ascertain whether the subjects had retained their diabetic status. Nonetheless, in the STZ-induced diabetes model, numerous studies have demonstrated that hyperglycemia can persist for an extended duration following a singular intraperitoneal administration of STZ [24]. Furthermore, the fundamental aims of the pharmacological agents employed in this study were to examine the protective mechanisms at play within cardiac tissue, rather than the modulation of glycemic levels. SGLT2-i’s cardiovascular effects were demonstrated to be independent of hyperglycemia in various clinical and preclinical investigations [7,27,28,67,68,69]. Conversely, trimetazidine exerts its effects by modulating energy metabolism, alleviating oxidative stress and inflammation, and providing benefits that are independent of blood glucose [54]. In future studies, measurements such as insulin, glucose, and c-peptide must be incorporated into the analyses to clarify whether the effects of these pharmacological agents on cardiotoxicity are mediated through glucose-dependent pathways. Another limitation of this study is the absence of pre-treatment biomarker measurements. While post-treatment comparisons were informative, future studies should incorporate pre–post assessments to better evaluate within-subject changes and treatment effects. Although histopathological and immunohistochemical analyses were performed by two independent, blinded researchers, a formal inter-rater reliability assessment (e.g., Cohen’s kappa or intraclass correlation coefficient) was not conducted. While discussions resolved scoring discrepancies, future studies may benefit from implementing formal inter-rater reliability measures to further enhance objectivity in subjective outcome assessments. Lastly, the findings obtained from the rat model cannot be fully generalized to the relationship between cardiotoxicity and diabetes in humans. Therefore, long-term experimental clinical studies on human subjects are needed.

## 5. Conclusions

In this study, the effects of trimetazidine and dapagliflozin, both independently and in combination, on cardiac tissue were examined immunohistochemically through ER stress in a rat model where cardiotoxicity was induced by doxorubicin and diabetes was induced by streptozotocin. The results of the histological and immunohistochemical analyses demonstrated that both drugs reduced ER stress and exhibited protective effects against cardiotoxicity. Combination therapy or trimetazidine alone was found to be slightly more effective than dapagliflozin in alleviating ER stress. Combination therapy offers promising potential for reducing cardiac cell damage caused by ER stress.

## Figures and Tables

**Table 1:** Histopathological cardiac damage scoring (HCDS).

Score	Findings
Degenerative cardiomyocyte	
0	≤5%
1	6–25%
2	26–50%
3	>50%
Vascular congestion	
0	≤5%
1	6–25%
2	26–50%
3	>50%
Edematous area	
0	≤5%
1	6–25%
2	26–50%
3	>50%

**Table 2:** Immunohistochemical (IHC) positivity scoring.

Score	Finding Cell Distribution That Shows IHC Positivity
0	≤5%
1	6–25%
2	26–50%
3	˃50%

**Table 3:** Biochemical levels of MDA and GSH in rat heart tissue.

Group	MDA (TBARS) (nmol/g Tissue)	GSH (TT) (mmol/g Tissue)
Control	17.57 ± 1.11	8.93 ± 0.48
STZ	24.22 ± 4.12	8.95 ± 1.24
STZ + DOXO	18.82 ± 3.02	9.3 ± 1.66
STZ + DOXO + TMZ	17.58 ± 3.34	10.3 ± 1.88
STZ + DOXO + DAPA	19.52 ± 4.00	8.68 ± 1.41
STZ + DOXO + TMZ + DAPA	19.68 ± 4.98	9.28 ± 1.27
* *p*-value > 0.05		

* No statistically significant difference was found between groups with One-Way ANOVA (*p* > 0.05). Abbreviations: MDA, malondyaldehyde; TBARS, Thiobarbituric Acid Reactive Substances; GSH, Glutathione; TT, total thiol; STZ, streptozotocine; DOXO, doxorubicin; TMZ, trimetazidine; DAPA, dapagliflozin.

**Table 4:** Histopathological cardiac damage scoring (HCDS).

Group	Degenerative Cardiomyocyte	Vascular Congestion	Edema	HCDS
Control	0 (0–0)	0 (0–0)	0 (0–0)	0 (0–1)
STZ	2 (2–2) ^a^	2 (2–2) ^a^	1 (1–1) ^a^	5 (5–5) ^a^
STZ + DOXO	3 (2–3) ^a,b,d^	3 (2–2) ^a,b,d^	1 (1–1) ^a^	6 (5–6) ^a,e^
STZ + DOXO + TMZ	1 (1–1) ^a,b,c^	1 (1–1) ^a,b,c^	1 (1–1) ^a^	3 (3–4) ^a,e^
STZ + DOXO + DAPA	1 (1–1) ^a,b,c^	1 (1–1) ^a,b,c^	1 (1–1) ^a^	3 (3–3) ^a,e^
STZ + DOXO + TMZ + DAPA	1 (1–1) ^a,b,c^	1 (1–1) ^a,b,c^	1 (0–1) ^a,e^	3 (3–3) ^a,e^
(median (25th–75th percentiles)). Kruskal–Wallis and Tamhane’s T2 test				

^a^ *p* = 0.001: compared with control group; ^b^ *p* = 0.001: compared with STZ group; ^c^ *p* = 0.001: compared with STZ + DOXO group; ^d^ *p* = 0.001: compared with STZ + DOXO + TMZ group; ^e^ *p* = 0.005: compared with control group. Abbreviations: HCDS, histopathological cardiac damage scoring; STZ, streptozotocine; DOXO, doxorubicin; TMZ, trimetazidine; DAPA, dapagliflozin.

**Table 5:** Immunohistochemistry positivity scoring results.

Group	CHOP Positivity Score	GRP 78 Positivity Score
Control	0 (0–1)	0 (0–0)
STZ	1 (1–2) ^a^	1 (1–2) ^a^
STZ + DOXO	2 (2–2) ^a,b,d^	1 (1–2) ^a^
STZ + DOXO + TMZ	1 (1–1) ^a,c^	0 (0–1) ^e,c^
STZ + DOXO + DAPA	1 (1–1) ^a,c^	1 (0–1) ^f,c^
STZ + DOXO + TMZ + DAPA	1 (1–1) ^a,c^	0 (0–1) ^b,c^
(median (25th–75th percentiles)). Kruskal–Wallis and Tamhane’s T2 test		

^a^ *p* = 0.001: compared with control group; ^b^ *p* = 0.001: compared with STZ group; ^c^ *p* = 0.001: compared with STZ + DOXO group; ^d^ *p* = 0.001: compared with STZ + DOXO + TMZ group; ^e^ *p* = 0.002: compared with STZ group; ^f^ *p* = 0.005: compared with STZ group. Abbreviations: STZ, streptozotocine; DOXO, doxorubicin; TMZ, trimetazidine; DAPA, dapagliflozin.

## Data Availability

Original data supporting the findings of this study are available. No copyright permissions are required for the figures in this study.
